# Predictive effect of triglyceride-glucose index on No-Reflow Phenomenon in patients with type 2 diabetes mellitus and acute myocardial infarction undergoing primary percutaneous coronary intervention

**DOI:** 10.1186/s13098-024-01306-y

**Published:** 2024-03-14

**Authors:** Juan Ma, Mohan Wang, Peng Wu, Xueping Ma, Dapeng Chen, Shaobin Jia, Ning Yan

**Affiliations:** 1 School of Clinical Medicine, Ningxia Medical University, 750004 Yinchuan, People’s Republic of China; 2 Heart Centre, Department of Cardiovascular Diseases, General Hospital of Ningxia Medical University, 750004 Yinchuan, Ningxia People’s Republic of China

**Keywords:** Triglyceride-glucose index (TyG index), Type 2 diabetes mellitus (T2DM), Acute myocardial infarction (AMI), No-reflow

## Abstract

**Objective:**

Triglyceride glucose (TyG) index is considered as a new alternative marker of insulin resistance and a clinical predictor of type 2 diabetes mellitus (T2DM) combined with coronary artery disease. However, the prognostic value of TyG index on No-Reflow (NR) Phenomenon in T2DM patients with acute myocardial infarction (AMI) remains unclear.

**Methods:**

In this retrospective study, 1683 patients with T2DM and AMI underwent primary percutaneous coronary intervention (PCI) were consecutively included between January 2014 and December 2019. The study population was divided into two groups as follows: Reflow (*n* = 1277) and No-reflow (*n* = 406) group. The TyG index was calculated as the ln [fasting triglycerides (mg/dL)×fasting plasma glucose (mg/dL)/2].Multivariable logistic regression models and receiver-operating characteristic curve analysis were conducted to predict the possible risk of no-reflow. Net Reclassification Improvement (NRI) and Integrated Discrimination Improvement (IDI) were calculated to determine the ability of the TyG index to contribute to the baseline risk model.

**Results:**

Multivariable logistic regression models revealed that the TyG index was positively associated with NR[OR,95%CI:5.03,(2.72,9.28),*p*<0.001] in patients with T2DM and AMI. The area under the curve (AUC) of the TyG index predicting the occurrence of NR was 0.645 (95% CI 0.615–0.673; *p* < 0.001)], with the cut-off value of 8.98. The addition of TyG index to a baseline risk model had an incremental effect on the predictive value for NR [net reclassification improvement (NRI): 0.077(0.043to 0.111), integrated discrimination improvement (IDI): 0.070 (0.031to 0.108), all *p* < 0.001].

**Conclusions:**

High TyG index was associated with an increased risk of no-reflow after PCI in AMI patients with T2DM. The TyG index may be a valid predictor of NR phenomenon of patients with T2DM and AMI. Early recognition of NR is critical to improve outcomes with AMI and T2DM patients.

## Introduction

Acute myocardial infarction (AMI) has been clearly considered the leading cause of cardiovascular morbidity and mortality worldwide [1].The World Bank assessed that the number of patients with AMI in China will increase to 23 million by 2030 [2]. Currently, primary percutaneous coronary artery intervention (PCI) remains the preferred method of treatment for individuals with AMI. However, No-reflow phenomenon (NR), which affects approximately 10–30% of patients, is characterized as the inadequate myocardial perfusion despite the mechanical reopening of the occluded artery post PCI [3]. Moreover, The detailed pathogenesis of NR remains unclear, but NR is an independent predictor of heart failure (HF), stroke, malignant arrhythmias, and in-hospital mortality [4]. Studies have shown that patients with type 2 diabetes mellitus(T2DM) combined with AMI are classified as high-risk group for NR, because they are more complex coronary artery disease [5]. Therefore, early recognition of residual risk factors in AMI patients with T2DM is essential for better clinical management to reduce the incidence of NR.

Insulin resistance (IR) refers to the decreased sensitivity of the body to insulin, not only involved in the pathogenesis of cardiovascular diseases but also significantly increased the incidence of adverse cardiovascular outcomes [6]. Hyper-insulinemic glucose clamp is a “gold standard” method to assess insulin sensitivity, but it is time-consuming and expensive and has limited clinical use [7]. The triglyceride–glucose (TyG) index was a parameter derived from the fasting plasma glucose (FBG) and triglyceride (TG) levels, which has been regarded as a convincing and substitute indicator of IR [8]. Some observational studies suggest that an elevated TyG index is associated with incident cardiovascular disease (CVD) [9]and poor CVD outcomes [10]. However, no trials have focused exclusively on TyG index prediction for NR in AMI patients with T2DM. The aim of our study was to fill this gap in knowledge.

## Methods

### Study population

**Fig. 1:**
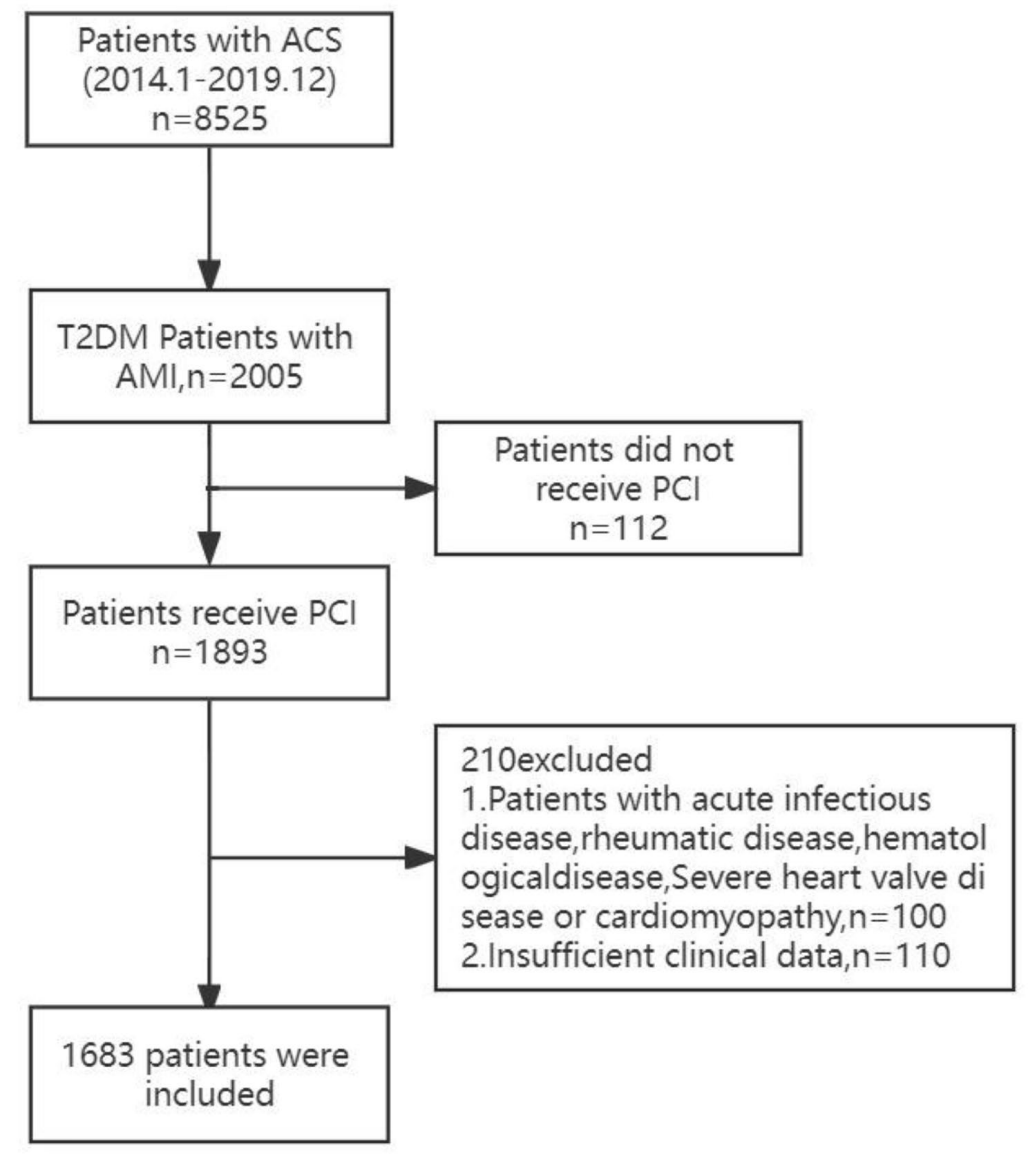
The flow chart of research subject. ACS, acute coronary syndrome; AMI, acute myocardial infarction; PCI, primary percutaneous coronary intervention; T2DM, type 2 diabetes mellitus

The study subjects were from the Department of Cardiovascular Medicine, General Hospital of Ningxia Medical University. The patient flow chart is shown in Fig. 1. A total of 8525 patients who were diagnosed with acute coronary syndrome (ACS) in the Department of Cardiovascular Medicine of General Hospital of Ningxia Medical University from January 2014 to December 2019 were selected. Of these 8525, 2005 were diagnosed with T2DM combined with AMI and underwent PCI. Of the 2005 patients, exclusion criteria were (1) acute infectious disease, rheumatic disease, hematologic disease, or (2) severe heart valve diseases or cardiomyopathy; and (3) insufficient clinical data. Finally, 1683 patients were included in this study. Based on the median value of the TyG index, 1683 patients were divided into two groups (TyG index < 8.76 group, *n* = 842 and TyG index ≥ 8.76 group, *n* = 841). Based on the Thrombolysis in Myocardial Infarction (TIMI) score,1683 patients were divided into two groups as follows: Reflow (*n* = 1277) and No-reflow (*n* = 406) group.

### Data collections and definitions

This study was approved by the Ethics Committee of General Hospital of Ningxia Medical University (number 2020 − 774).

Patient demographics, past medical history, examination test results, and data related to echocardiography and angiography were collected through the electronic medical record system.

Criteria for T2DM include (1) a previous diagnosis of T2DM on antidiabetic medication; (2) typical diabetic symptoms (excessive thirst and drinking, polyuria, polyphagia, and unexplained weight loss) with an FPG ≥ 7.0 mmol/L, and/or a randomized glucose ≥ 11.1 mmol/L, and/or a plasma glucose level ≥ 11.1 mmol/L at 2 h post OGTT, and/or glycosylated hemoglobin (HbA1c) ≥ 6.5%.AMI was categorized as ST-segment elevation myocardial infarction (STEMI)and No-ST-segment elevation myocardial infarction (NSTEMI), which was defined as chest pain accompanied by new ST-segment changes, concurrent elevated cardiac troponin values with at least one value above the upper 99th percentile reference limit. Fasting TG and FPG were fasting blood concentrations taken for the first time after the patient had abstained from eating for at least 10 h during hospitalization. The TyG index was calculated by the formula ln [fasting TG (mg/dL) × FPG (mg/dL)/2] [11].

### Percutaneous coronary angiography and definition of post-operative no-reflow

All AMI patients underwent primary PCI. Pre-operative pharmacological treatment consisted of 300 mg aspirin and 180 mg of Ticagrelor or 300–600 mg Clopidogrel according to the clinical guidelines. PCI was decided and performed by two specialists according to the patient’s actual condition of the vessel lesion, and the Thrombolysis in Myocardial Infarction (TIMI) score was recorded according to the results of coronary angiography to assess the coronary blood flow after PCI. Specifically, TIMI grades 0, 1and 2 were defined as no-reflow and TIMI grades 3 as reflow after excluding mechanical conditions such as coronary spasm and occlusion [12]. Patients with NR are usually treated clinically with adenosine, calcium channel blockers, sodium nitroprusside, glycoprotein IIb/IIIa inhibitors or combinations of these drugs.

### Statistical analysis

Empower Stats version 3.0 (http://www.empowerstats.com) and the software packages R version 3.4.3 (http://www.R-project.org) were applied for statistical analysis. Continuous variables were presented as mean ± standard deviation (SD) or the lower and upper quartile values (25th, 75th). Student’s t-test or Mann-Whitney U test was used to analysis comparisons between the 2 study groups. Categorical variables were expressed as numbers and percentages. Comparisons were made using the Pearson chi-squared test or Fisher’s exact test. The multivariate model included baseline variables that were significantly correlated with NR on univariate analysis and were clinically relevant. In addition, intercorrelations between variables were also taken into account in the multivariate analysis. Receiver-operating characteristic (ROC) curve analysis was performed to determine the optimal cut-off point value of TyG index for predicting NR. Net Reclassification Improvement (NRI) and Integrated Discrimination Improvement (IDI) were calculated to determine the extent to which the addition of the TyG index improved the predictive ability of the of the existing baseline risk model. A two-tailed *p* value<0.5 was considered as statistically significant.

## Results

### Baseline clinical characteristics of patients

Baseline clinical characteristics of the total population and groups stratified by with or without NR were presented in Table 1. Of 1683 T2DM patients with AMI,71.89% (*n* = 1210) were STEMI and 28.1%(*n* = 473) were NSTEMI.67.91% (*n* = 1143) were male, and 32.08% (*n* = 540 patients) were female, and 406 (*n* = 24.12%) patients developed NR following PCI. The TyG index level and the proportion of the patients with TyG ≥ 8.79 was significantly higher in NR group than that in the reflow group. Patients with NR showed higher age, white blood cells(WBC), creatinine, triglyceride(TG),FPG, higher proportion of female, initial TIMI and higher prevalence of CAD history. However, patients in NR group had lower levels of diastolic blood pressure (DBP), hemoglobin, estimated glomerular filtration rate(eGFR), left ventricular ejection fraction (LVEF) and lower prevalence of smoking history. In terms of the angiographic findings, those with NR showed lower proportions of thrombus aspiration, left anterior descending (LAD) but higher proportions of right coronary artery (RCA). In addition, The proportion treated with statins was lower in patients with NR.

**Table 1:** Baseline clinical characteristics of the patients stratified by NR

Variables	Reflow (*n* = 1277)	No-reflow(*n* = 406)	*P* value
TyG index			<0.001
TyG<8.79	704(55.13%)	138(33.99%)	
TyG ≥ 8.79	573(44.87%)	268(66.01%)	
SPISE index	6.92 ± 1.75	6.92 ± 1.78	0.576
Age, years	61.80 ± 10.52	67.37 ± 10.56	< 0.001
Female gender	374(29.29%)	166(40.89%)	< 0.001
BMI	24.86 ± 4.58	24.24 ± 4.08	0.056
SBP	125.32 ± 22.62	122.79 ± 23.51	0.080
DBP	76.82 ± 13.74	73.94 ± 14.14	0.002
Medical history			
Hypertension	856(67.03%)	290(71.43%)	0.098
Dyslipidemia	567(44.40%)	155(38.18%)	0.256
Current smoking	678 (53.13%)	194 (47.78%)	0.039
Previous CAD	249 (19.50%)	124 (30.54%)	< 0.001
Laboratory values			
WBC,109/L	10.04 ± 3.72	10.70 ± 4.33	0.032
Neutrophil count, 109/L	40.65 ± 33.92	41.88 ± 35.09	0.261
Lymphocyte count,109/L	10.19 ± 11.13	9.30 ± 10.34	0.157
Monocyte count,109/L	3.34 ± 3.38	3.63 ± 3.72	0.140
Hemoglobin, g/L	109.64 ± 58.25	89.11 ± 58.87	< 0.001
Platelet count,109/L	221.60 ± 64.25	223.63 ± 76.07	0.598
HbA1c, %	8.13 ± 1.80	8.15 ± 1.80	0.829
Creatinine, umol/L	76.95 ± 41.69	105.72 ± 102.85	< 0.001
eGFR,ml/min/1.73m2	94.84 ± 38.86	71.45 ± 35.65	< 0.001
TG, mmol/L	2.03 ± 1.58	2.47 ± 1.39	< 0.001
TC, mmol/L	4.05 ± 1.06	3.96 ± 0.96	0.725
LDL-C, mmol/L	2.14 ± 0.75	2.16 ± 0.88	0.628
HDL-C, mmol/L	0.88 ± 0.22	0.90 ± 0.26	0.439
FPG, mmol/L	11.09 ± 4.77	12.65 ± 5.60	< 0.001
Echocardiography			
LVEF,%	52.50 ± 10.27	46.99 ± 12.44	< 0.001
AMI			0.348
STEMI	922 (72.20%)	288 (70.94%)	
NSTEMI	355 (27.80%)	118 (29.06%)	
Angiographic characteristics			
Initial TIMI			<0.01
0	615 (48.16%)	367 (90.39%)	
1	71 (5.56%)	6 (1.48%)	
2	137 (10.73%)	9 (2.22%)	
3	454 (35.55%)	24 (5.91%)	
Thrombus aspiration	81 (6.34%)	14 (3.45%)	0.028
Infarction related artery			
LM	13 (1.02%)	1 (0.25%)	0.136
LAD	604 (47.30%)	44 (10.84%)	< 0.001
RCA	483 (37.82%)	301 (74.14%)	< 0.001
LCX	190 (14.88%)	61 (15.02%)	0.943
Number of stents	1.35 ± 0.68	1.13 ± 0.43	0.243
Stent diameter, mm	3.05 ± 0.45	3.28 ± 0.46	0.183
Stent length, mm	26.88 ± 8.12	28.81 ± 7.92	0.164
IABP	28 (2.19%)	4 (0.99%)	0.121
Calcifying lesion	29 (2.27%)	5 (1.23%)	0.195
Treatments			
Aspirin	1275 (99.84%)	404 (99.51%)	0.247
Ticagrelor	653 (51.14%)	210 (51.72%)	0.836
Clopidogrel	622 (48.71%)	196 (48.28%)	0.218
Beta-blocker	981 (76.82%)	311 (76.60%)	0.927
Statin	1267 (99.22%)	386 (95.07%)	< 0.001
ACEI/ARB	635 (49.73%)	200 (49.26%)	0.223

Values are presented as mean ± SD, or number (%),or median(interquartile range). Abbreviations: TyG, triglyceride-glucose index; TG, triglyceride; HDL-C, high-density lipoprotein cholesterol; SPISE index, the Single Point Insulin Sensitivity Estimator; BMI, body mass index; SBP, systolic blood pressure; DBP, diastolic blood pressure; CAD, coronary artery disease;AMI, myocardial infarction; WBC, white blood cell; FPG, fasting plasma glucose; HbA1c, glycated hemoglobin; eGFR, estimated glomerular filtration rate; TC, total cholesterol; LDL-C, low-density lipoprotein cholesterol; LVEF, left ventricular ejection fraction; LM, left main coronary artery; LAD, left anterior descending; LCX, left circumflex coronary artery; RCA, right coronary artery; ACEI/ARB, angiotensin-converting enzyme inhibitor/angiotensin receptor blocker.

### TyG index predicted the occurrence of NR

Univariate and multivariate logistic regression analysis and predictors for NR were presented in Table 2. Univariate analysis showed TyG index, age, female, previous history of CAD, DBP, WBC, Hemoglobin, TG, FPG, Creatinine, eGFR, LVEF, Thrombus aspiration, Statin, LAD and RCA coronary artery lesions were risk factors for NR in T2DM patients with AMI (all *p* < 0.05). The results of co-linearity analysis of NR predictors and TyG index are revealed in Table 3. In addition, TyG was significantly related with TG (*r* = 0.8284, *p* < 0.001) and FPG (*r* = 0.6048, *p* < 0.001). eGFR was significantly correlated with creatinine (*r*=-0.6486, *p* < 0.001). Therefore, TG, FPG, and creatinine were also not included in the multivariate analysis. Therefore, multivariate analysis found that the TyG index, age, LVEF, statin and LAD and RCA coronary artery lesions were independent predictors of NR in T2DMpatients with AMI (all *p* < 0.05, Table 2).

**Table 2:** Results of univariate and multivariate analysis of NR.

	Univariate		Multivariate	
	OR (95%CI)	*P* value	OR (95%CI)	*P* value
Age	1.05 (1.04, 1.07)	< 0.0001	1.03 (1.02, 1.05)	0.0003
Female	1.67 (1.32, 2.11)	< 0.0001	1.02 (0.73, 1.42)	0.9185
Current smoking	0.81 (0.65, 1.01)	0.0665		
Previous CAD	1.82 (1.41, 2.34)	< 0.0001	1.31 (0.94, 1.82)	0.1109
DBP	0.99 (0.98, 0.99)	0.0003	0.99 (0.98, 1.00)	0.1589
WBC	1.04 (1.01, 1.07)	0.0031	1.03 (0.99, 1.07)	0.1011
Hemoglobin	0.99 (0.99, 1.00)	< 0.0001	1.00 (1.00, 1.00)	0.2597
TG	1.18 (1.10, 1.27)	< 0.0001		
TyG	1.96 (1.68, 2.29)	< 0.0001	5.03 (2.72, 9.28)	< 0.0001
FPG	1.06 (1.04, 1.08)	< 0.0001		
Creatinine	1.01 (1.01, 1.01)	< 0.0001		
eGFR	0.98 (0.98, 0.99)	< 0.0001	1.00 (0.99, 1.00)	0.5223
LVEF	0.95 (0.94, 0.97)	< 0.0001	0.96 (0.94, 0.97)	< 0.0001
Statin	6.43 (4.03, 10.25)	< 0.0001	8.81 (5.09, 15.25)	< 0.0001
Thrombus aspiration	0.53 (0.30, 0.94)	0.0302	0.63 (0.33, 1.22)	0.1696
LAD	0.14 (0.10, 0.19)	< 0.0001	0.24 (0.15, 0.40)	< 0.0001
RCA	4.71 (3.67, 6.05)	< 0.0001	2.61 (1.76, 3.88)	< 0.0001

Abbreviations: TyG, triglyceride-glucose index; DBP, diastolic blood pressure; CAD, coronary artery disease; WBC, white blood cell; TG, triglyceride; FPG, fasting plasma glucose; eGFR, estimated glomerular filtration rate; LVEF, left ventricular ejection fraction; LAD, left anterior descending; RCA, right coronary artery. OR, odds ratio; CI, confidence interval.

**Table 3:** Co-linearity analysis of NR predictors and TyG index

	Unstandardized coefficients				Collinearity statistics
	B	Std, error	Exp(B)	*P* value	VIF
Age	0.052	0.006	1.054	< 0.0001	1.900
Female	0.513	0.118	1.670	< 0.0001	1.700
Current smoking	-0.210	0.114	0.820	0.0665	1.500
Previous CAD	0.596	0.129	1.815	< 0.0001	1.100
DBP	-0.015	0.004	0.985	0.0003	1.100
WBC	0.042	0.014	1.043	0.0031	1.100
Hemoglobin	-0.006	0.010	0.994	< 0.0001	1.100
TG	0.168	0.036	1.184	< 0.0001	4.500
FPG	0.058	0.011	1.060	< 0.0001	2.900
Creatinine	0.008	0.001	1.008	< 0.0001	1.500
eGFR	-0.018	0.002	0.983	< 0.0001	2.400
LVEF	-0.046	0.006	0.9550	< 0.0001	1.200
Statin	1.8604	0.2383	6.4266	< 0.0001	1
Thrombus aspiration	-0.6399	0.2952	0.5273	0.0302	1
LAD	-1.9993	0.1692	0.1354	< 0.0001	2.3
RCA	1.5502	0.1272	4.7125	< 0.0001	2.3

Dependent variable: TyG index. Abbreviations: TyG, triglyceride-glucose index; DBP, diastolic blood pressure; CAD, coronary artery disease; WBC, white blood cell; TG, triglyceride; FPG, fasting plasma glucose; eGFR, estimated glomerular filtration rate; LVEF, left ventricular ejection fraction; LAD, left anterior descending; RCA, right coronary artery.

### ROC curve analyses to predict NR

**Fig. 2:**
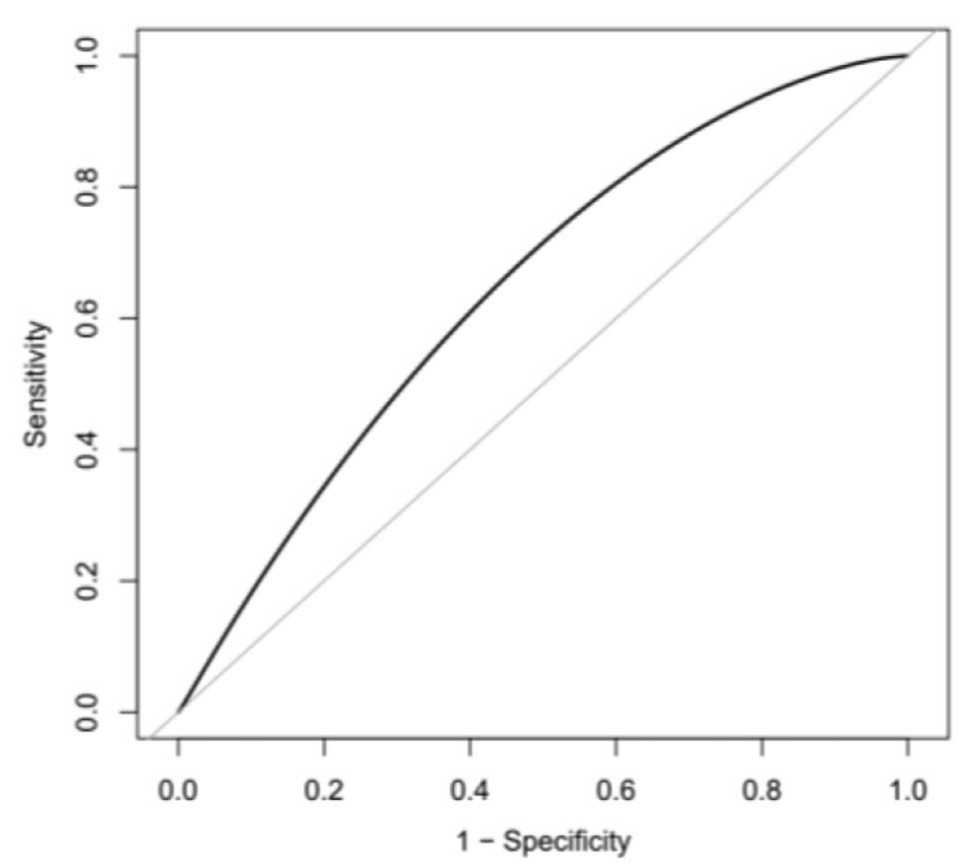
The receiver operating characteristic (ROC) curves of the TyG index as a marker to predict NR in patients with T2DM and AMI. The area under ROC curve (AUC) of the TyG index for predicting the occurrence of NR was 0.645 (95% CI 0.615–0.673; *p* < 0.001). The cut-of value of TyG index to predict NR was 8.98, the sensitivity was 0.571, and the specifcity was 0.667

The area under the ROC curve (AUC) for the TyG index to predict the incidence of NR phenomenon was 0.645 (95% CI 0.615–0.673; *p* < 0.001) (Fig. 2). The cut-of value of TyG index to predict NR was 8.98, the sensitivity was 0.571, and the specificity was 0.667.

### Incremental effect of TyG index on predictive value for NR

Table 4 showed that compared with the FPG and TG, the addition of TyG index significantly improved the reclassification and discrimination ability beyond the baseline risk model with NRI of 0.077and IDI of 0.070 (both *p* < 0.001).

**Table 4:** Evaluate the incremental predictive value and predictive power of various models with NRI and IDI

	Category-free NRI			IDI		
	index	95%CI	*P* value	index	95%CI	*P* value
Baseline risk model			Ref			Ref
+FPG	0.037	0.018 to 0.055	<0.001	0.009	-0.013 to 0.030	0.427
+TG	-0.039	-0.065 to-0.013	0.004	0.031	-0.001to 0.063	0.053
+TyG	0.077	0.043 to 0.111	<0.001	0.070	0.031 to 0.108	<0.001

Baseline risk model including age, LVEF, in-hospital statins and LAD and RCA coronary artery lesions. Abbreviations: NRI, net reclassification improvement; IDI, integrated discrimination improvement; FPG, fasting plasma glucose; TG, triglyceride; TyG, triglyceride-glucose index; LVEF, left ventricular ejection fraction; LAD, left anterior descending; RCA, right coronary artery; Ref, reference.

## Discussion

This article is mainly a study to evaluate the relationship between TyG index and NR in AMI patients with T2DM. To our knowledge, the novel point of this study is to show the association between the TyG index and NR in AMI patients with T2DM. Our major findings include:(1) the occurrence of NR increased significantly with the increase of TyG index, and (2) the TyG index was an independent predictor of NR, and (3) The AUC of the TyG index for the prediction of the occurrence of NR was 0.645 with a cut-off 8.98 and (4) adding the TyG index to a baseline risk model had an incremental effect on the predictive value of NR. Based on this study, we confirmed that the TyG index was positively associated with the occurrence of NR. Most importantly, this study demonstrated that a simple method to estimate IR may optimize risk stratification for the occurrence of NR in AMI patients with T2DM.

IR is defined as a decreased ability of insulin to promote glucose utilization and uptake and is an indicator of abnormal glucose and fat metabolism. And IR contributes to the progression of cardiovascular diseases by inducing imbalances in glucose metabolism, altering systemic lipid metabolism and endothelial cell dysfunction [13]. Several previous studies have confirmed that IR is an important risk factor for cardiovascular disease [14] and adverse clinical outcomes [15]. Currently, the classic methods for detecting IR include the hyper-insulinemic euglycemic clamp and HOMA-IR [16]. However, due to the complexity and high cost of the detection steps, the above two methods cannot be widely used in clinical practice. To address this clinical challenge, researchers have conducted extensive studies on the TyG index and found that the TyG index is a reliable alternative indicator of IR [17]. Therefore, the TyG index can be used in clinical practice to identify IR when the hyper-insulinemic euglycemic clamp test and HOMA-IR are not measurable.

Numerous studies have shown the ability of the TyG index to predict CVDs. Da Silva et al. found a positive correlation between the TyG index and the prevalence of coronary heart disease in patients on secondary CVD prevention [18]. A cohort study by Luo E et al. showed that in 1092 patients with STEMI treated by PCI, patients with TyG ≥ 9.608 had an increased risk of composite adverse cardiovascular events and all-cause mortality at 30 days, 6 months, and 1 year, and TyG ≥ 9.608 was independently associated with an increased risk of composite adverse cardiovascular events at 1 year [HR (95% CI) 1.53 (1.0, 2.06), *p* = 0.003] [19]. In addition, a study of 798 NSTEMI patients with T2DM treated with PCI suggested that a 1-unit increase in the TyG index was independently associated with an increased risk of the composite adverse cardiac and cerebral events [HR: 3. 208 / 1 unit, 95% CI 2.40–4.29, *p* < 0.001] [20]. However, the predictive effects of the TyG index on NR in AMI patients with T2DM are still unclear.

The no-reflow phenomenon is the Achilles heel of primary percutaneous coronary intervention. Studies have shown that NR is a strong and independent predictor of adverse cardiovascular outcomes, including acute heart failure, cardiogenic shock and life-threatening arrhythmias in patients with AMI [21], although the underlying molecular mechanisms are complex and poorly understood, several causative mechanisms are implicated, including endothelial dysfunction, oxidative stress, inflammation, microvascular injury and reperfusion injury [22].

The study has shown that in AMI patients with comorbid T2DM, the longer the duration of diabetes, the higher the preoperative glucose level, the slower the coronary angiographic blood flow, the narrower the coronary arteries, and the greater the likelihood of no-reflow after PCI [23]. Insulin can mediate anti-lipolysis, but IR in adipose tissue can reduce this effect, leading to a decrease in lipoprotein lipase activity and further induction of hyperlipidemia, it is a major cause of dyslipidemia in T2DM and can aggravate coronary atherosclerosis [24]. Additionally, Hyperglycemia in patients with AMI may further contribute to the phenomenon of no-reflow by increasing leukocyte obstruction in the capillaries, leading to increased levels of intercellular adhesion molecule-1 or p-selectin [25]. Micro-thrombosis after AMI has been shown to play an important role in the prevention of reflow obstruction. Hyperglycemia is an independent predictor of platelet-dependent thrombosis by exacerbating platelet-dependent thrombosis [26], increasing circulating adhesion molecules and capillary leukocyte occlusion, attenuating endothelium-dependent vasodilation and reducing collateral blood flow by impairing nitric oxide availability [27]. Furthermore, hyperglycemia attenuates ischemic preconditioning by reducing activation of mitochondrial adenosine triphosphate-regulated potassium channels, an independent predictor of the anemia phenomenon [28].

In addition, our data suggested that statin use was lower in the NR group than in the reflow group, and statins may reduce the incidence of NR. This result is consistent with the conclusion of Zhou et al [29]. Although the mechanism for this is not fully understood, it may result from improved endothelial function, inhibition of thrombogenic function, improved stability of atherosclerotic plaques, and reduced oxidative stress and vascular inflammation [30]. In the acute phase of AMI, statins protect the heart by raising endothelial nitric oxide levels by upregulating endothelial nitric oxide synthase, which is important for vasodilation, endothelial leukocyte interactions, vascular smooth muscle proliferation and platelet aggregation [31]. Statins exert their anti-inflammatory effects by reducing inflammatory cytokines such as interleukins and inhibiting pro-inflammatory biological processes such as monocyte chemotaxis and nuclear factor-κB activation, given the established link between coronary atherosclerosis and inflammation [32]. Statins improve coronary microcirculation by multiple mechanisms.

In this study, we investigated the prognostic value of the TyG index in the absence of regurgitation during PCI in AMI patients with T2DM for the first time. To better understand the predictive ability of the TyG index for no-reflow, we analyzed the correlation between different levels of the TyG index and NR, and we examined whether the inclusion of the TyG index in the baseline risk model would have any additional effect on the predictive value of no-reflow, something that has not been tried in other studies.

### Study limitations

It is important to address the following limitations of the present study. First, the results should be replicated with caution, as the study was limited to a single center and the sample size was small. Second, there was no record of the duration of diabetes and the use of previous antihyperglycemic therapy in AMI patients. Third, the relationship between the TyG index and IR cannot be directly verified in this study because conventional laboratory tests for IR, such as HOMA-IR, were not tested.

In addition, a larger sample size and multi-center cohort studies are required to verify our conclusions.

## Conclusions

In conclusion, this study has demonstrated that elevated TyG index level was a strong independent predictor of no-reflow phenomenon in AMI patients with AMI. In addition, adding the TyG index to a baseline risk model had an incremental effect on the predictive value for no-reflow phenomenon.

## Data Availability

The datasets generated and/or analyzed during the current study are not publicly available due to the ongoing nature of this study but are available from the corresponding author on reasonable request.
